# Quantification of aniline and N-methylaniline in indigo

**DOI:** 10.1038/s41598-021-00634-7

**Published:** 2021-10-26

**Authors:** Michael Cordin, Thomas Bechtold, Tung Pham

**Affiliations:** Research Institute of Textile Chemistry and Textile Physics, University of Innsbruck, Hoechsterstrasse 73, 6850 Dornbirn, Austria

**Keywords:** Environmental sciences, Chemistry

## Abstract

Aniline and N-methylaniline are common contaminants in commercially produced indigo. It is known, that commercially produced indigo contains up to 0.6% aniline and 0.4% N-methylaniline by weight and indigo dye shows a small mutagenic effect, most probably due to the presence of these contaminants. The present work describes a new and powerful analytical method to determine the concentration of these contaminants in indigo. This method is based on the transformation of water insoluble indigo into soluble leucoindigo and allows therefore the acidic extraction of the aromatic contaminants. This transformation step is essential, because the main part of these contaminants are strongly included in the indigo crystals. The amount of extracted aniline and N-methylaniline from the leucoindigo solution was quantified with high performance liquid chromatography (HPLC, combined with a photo diode array detector). A possible accumulation of the aromatic amines at the indigo crystal surface was investigated using FTIR and by adsorption studies. Therefore this method allows an accurate monitoring of these toxic by-products in the indigo dye, which is important for an economic and environmental assessment of the denim production.

## Introduction

Indigo has a great economic importance for the dyeing of approximately 4 billion denim textiles per year. For this purpose 70,000 t of indigo are used and an amount between 10 and 20 million m^3^ water is released with residual chemicals from the dyeing step and by products from the indigo synthesis, i.e. aniline and N-methylaniline^1^.

**Figure 1:**
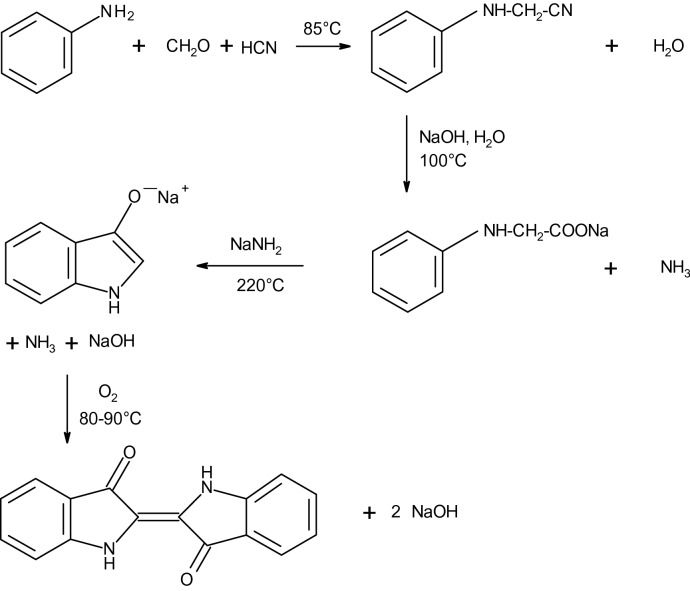
Principal synthesis route of indigo with aniline as starting material according to Pfleger and Heumann^3^.

**Figure 2:**
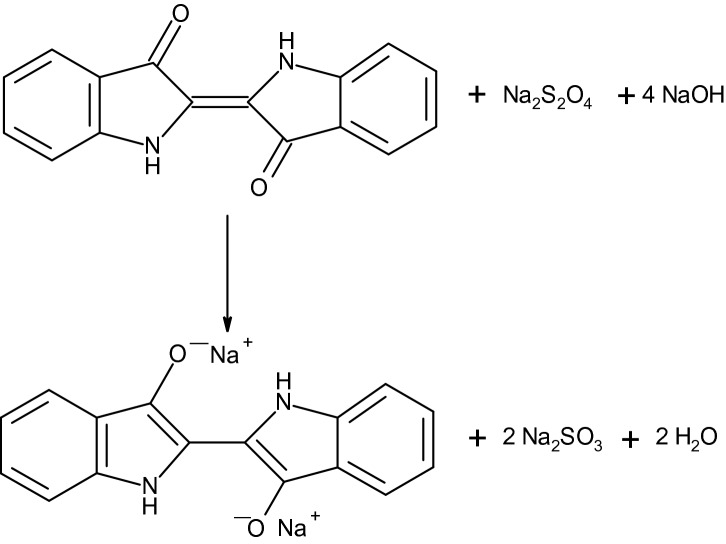
Reduction of indigo to leucoindigo with sodium dithionite.

Indigo (C.I. Vat Blue 1) is an important blue dye, which is known and used since antiquity^2^. Indigo containing plants were cultivated in many parts in the world, so the woad plant (*Isatis tinctoria*) in Northern Europe and the so called indigo plant (*Indigofera species*) in Asia. The quality of natural indigo varies dependent on the country of origin and harvest and therefore the reproducibility of the dyeing process was difficult. This situation changed with the advent of synthetic indigo. Already in 1870 Adolf von Baeyer showed, that indigo can be prepared by reduction of Isatin and later he enhanced this method to a full synthetic approach with phenylacetic acid as starting material^3,4^. The first technically suitable synthesis was developed by Karl Heumann in 1890, which was later improved by Johannes Pfleger in 1901 by the addition of sodium amide to the alkali melt. Subsequently the two German companies BASF and Hoechst produced synthetic indigo with this method, which is still used today with the principal synthesis route as shown in Fig. 1. The synthesis with aniline as starting material is combined with higher costs for the technical equipment, but the used raw materials are cheaper^4^. Later synthetic blue dyes were developed as alternatives for indigo with superior properties and therefore the production volume of indigo decreased overtime. The decline was stopped however by the hugely growing market of blue jeans. Indigo is water insoluble and is therefore not directly suitable for the dyeing process, but it can be transformed into leucoindigo by reduction and this substance is soluble in aqueous alkali. In former times this reduction was done by a fermentation process, consisting of carbohydrate based syrup with alkali, whereby the reducing agents were the carbohydrates. Later the reduction was replaced by more suitable substances, such as iron(II)-sulfate and zinc powder. Today the reduction is usually performed with sodium dithionite, the reaction is shown in Fig. 2. The reduction with sodium dithionite leads to the formation of substantial quantities of waste water and thus led to the development of alternative reduction methods, such as the reduction with glucose, catalytic hydrogenation and a direct or indirect electrochemical reduction with a reversible redox system as mediator^5–14^. Indigo sublimes above 170 °C and has a melting temperature of approximately 390–392 °C. The blue-violet indigo crystals are hardly soluble and are only slightly soluble in hot polar solvents, such as aniline or dimethyl sulfoxide. Indigo forms intra- and intermolecular hydrogen bonds, which are responsible for the high melting temperature and low solubility in most solvents and also influences the reflectance spectrum of indigo^15–20^.

By concentrated alkali and higher temperatures indigo can be decomposed to aniline, N-methylaniline and anthranilic acid^4^. It is known that the Pfleger-Heumann synthesis leads to some decomposition substances of indigo, such as aniline, N-methylaniline and anthranilic acid. Besides the intermediate phenylglycin nitrile already contains aniline and therefore a subsequent purification of crude indigo is necessary, which was done by steam distillation and later replaced by continuous solvent extraction^3^. These byproducts can also be found in the waste water, whereby aniline and anthranilic acid are more biodegradable than N-methylaniline. Synthetic indigo shows a small mutagenic effect, most probably due to the presence of these contaminants^21,22^. During the production of indigo and also during the dyeing of textiles, aniline and N-methylaniline can be released into the waste water. However, the US Environmental Protection Agency (EPA) has not defined a general Maximum Contaminant Level (MCL) for aniline in drinking water. The LC50 value for fish (Oncorhynchus mykiss) can be as low as 10.6 mg/L and shows clearly the danger for the environment by the release of aniline by dye plants^23^. The maximum concentration of aniline in the air at the workplace (MAK value) in Germany is fixed to 7.7 mg/m^3^.

According to the US Environmental Protection Agency (EPA), aniline has been classified to be very toxic in humans with an estimated oral lethal dose between 50 and 500 mg/kg. Short-term exposure to aniline can have a negative effect on the functioning of the lungs and chronic exposure can have an effect on the blood and is probably human carcinogen (as classified by EPA).

Attempts to purify indigo and to remove the main byproducts aniline and N-methylaniline are described in several patents^24–27^. Approaches to remove aniline and N-methylaniline by washing with acids, extraction with solvents or by steam distillation were reported to be not successful (and are therefore no appropriate extraction methods for their quantification). This leads to the conclusion that these substances are included in the indigo crystals and require therefore special analytical methods to prove the presence of these substances. Synthetic indigo usually contains up to 0.6% aniline and up to 0.4% by weight of N-methylaniline^24^. There are almost no reports in the scientific literature about these contaminants in indigo. However, there are efforts of indigo manufacturers to remove these contaminants^24–27^. There are attempts described in the scientific literature about the cleaning of indigo waste water, but these investigations do not include a possible removal (or degradation) of aniline and N-methylaniline from the waste water^28–31^. The presence of these contaminants in indigo shows clearly that a powerful analytical method to determine the concentration is very important. There are reports in the literature about principal methods (mainly based on HPLC) to quantify aniline in different environments^32–41^. Reports in the literature indicate, that the predominant method to quantify aniline and N-methylaniline in indigo is the photometric analytical technique^27^. However, this technique has significant disadvantages, as the indigo dye also contains other substances, which absorb at the chosen wavelength of measurement.

In this study a new analytical procedure to monitor aniline and N-methylaniline in indigo and leucoindigo solutions is presented. Following to an optimised extraction method the quantification of the released aniline and N-methylaniline is achieved by HPLC separation and UV-detection.

## Experimental

### Materials and sample preparation

**Figure 3:**
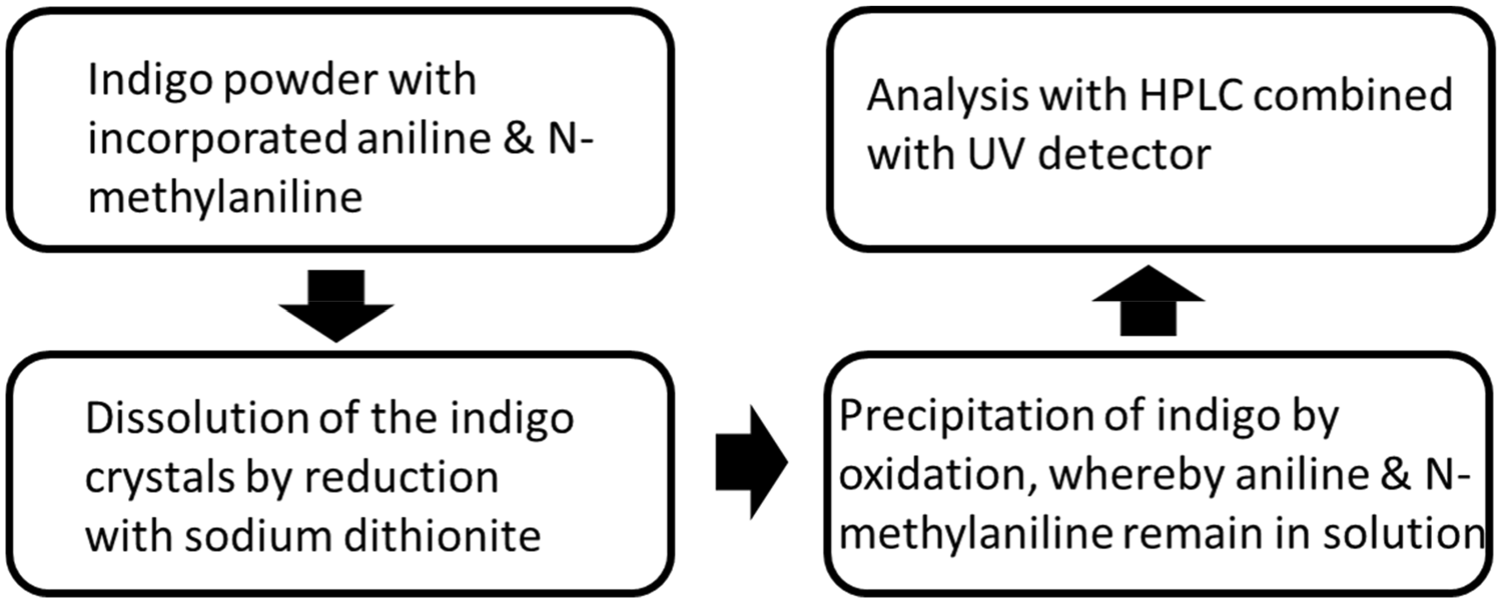
The basic procedure for the analysis of aniline and N-methylaniline in indigo with HPLC.

Technical-grade indigo samples were collected on the global market and provided by Blueconnection (Singapore). They were used to determine the aniline and N-methylaniline concentration. The analysed leucoindigo solutions contain 30% indigo by weight and alkali hydroxide in water and are protected against oxidation with an inert gas. Besides extraction experiments of solid indigo powder were performed. The basic procedure for the analysis of aniline and N-methylaniline is shown in Fig. 3. The key step for a successful quantification of the aromatic amines is the dissolution of the indigo crystals. This can be done by reduction of indigo to leucoindigo with an alkaline sodium dithionite solution (the reducing solution contains 4 g/L KOH and 12 g/L sodium dithionite and the reduction was performed at 70 °C). A defined part of the leucoindigo solution was added to a 0.01 M HCl solution and was then oxidized by bubbling air through the solution. As a consequence the leucoindigo is transformed to indigo, which precipitates. The resulting clear solution contains the aniline and N-methylaniline and is ready for the quantification with HPLC. The acidic extraction is working almost quantitatively by the transformation of insoluble indigo into alkaline water soluble leucoindigo. The efficiency of this method was shown by a second extraction of the same indigo, whereby almost no further aniline and N-methylaniline were extracted from the indigo.

The high performance liquid chromatography (HPLC) measurement was performed with a 250 mm long reversed phase silica-C18 column (Nucleosil, Macherey–Nagel). The particle size of the column is 5 µm and the pore size is 100 Å. An acetonitrile–water mixture with a volume fraction of 70 to 30 was prepared and used as eluent.

### Analytical methods

The HPLC measurements were performed with a Nexera XR device (Shimadzu, Japan), which is equipped with a refractive index- and UV-detector. During the HPLC measurement the column temperature was set to 30 °C, the pump performance was 0.7 mL/min, the injection volume was 10 μL and the LC stop time was 15 min. The UV-detector at 190 nm was used to quantify the aniline and N-methylaniline concentration. The detector showed the strongest detection signal for aniline and N-methylaniline at 190 nm (with respect to the baseline). Under these HPLC conditions the retention time of aniline was 4.94 min and that of N-methylaniline was 5.76 min. Consequently there is a clear difference in the retention time between aniline and N-methylaniline by the used eluent acetonitrile–water.

FTIR measurements were performed with the FT-IR microscope Lumos (Bruker, Germany). The microscope is equipped with a liquid N_2_ cooled MCT detector, providing a spectral range from 4000 to 600 cm^−1^ with a spectral resolution of 2 cm^−1^. The IR measurements were performed in the reflection mode.

### Validation of the HPLC method

#### Specificity

There is no overlap of the aniline (and N-methylaniline) HPLC-peaks with other substances—this was shown by the determination of the aniline (and N-methylaniline)-concentration with different detector wavelengths (between 190 and 800 nm). The concentration is (normally) only independent from the detector wave-length, if the HPLC peak is the result of a pure substance.

#### Linearity

It was shown, that the HPLC-measurement signal is directly proportional (R^2^ > 0.999) to the aniline and N-methylaniline concentration in a range between 0.010% and 1.5% (with regard to the indigo weight).

#### Quantitation limit

It was shown, that aniline and N-methylaniline can be confidently detected with a concentration as low as 0.010% (with regard to the indigo weight).

#### Accuracy

The aniline and N-methylaniline concentrations were determined by internal standard addition. The measurement signals of the sample with no internal standard and of three samples with varying amounts of added internal standard can be described with a fitted line. The coefficients of determination R^2^ of the fitted lines are better than 0.999.

It was possible to determine the concentration of a known added amount of aniline with an accuracy of 0.11% and that of N-methylaniline with an accuracy of 0.36%.

#### Precision of repeatability

The relative standard deviation of the repeatability for the determination of aniline and N-methylaniline concentration is better than 0.5%.

## Results and discussion

It is known, that the interaction between indigo and aniline molecules is relatively strong, because aniline is one of the few liquids, which is able to dissolve indigo at higher temperatures. During the technical synthesis of indigo, the concentration of aniline and the precursor molecules for the indigo formation is relatively high.

**Figure 4:**
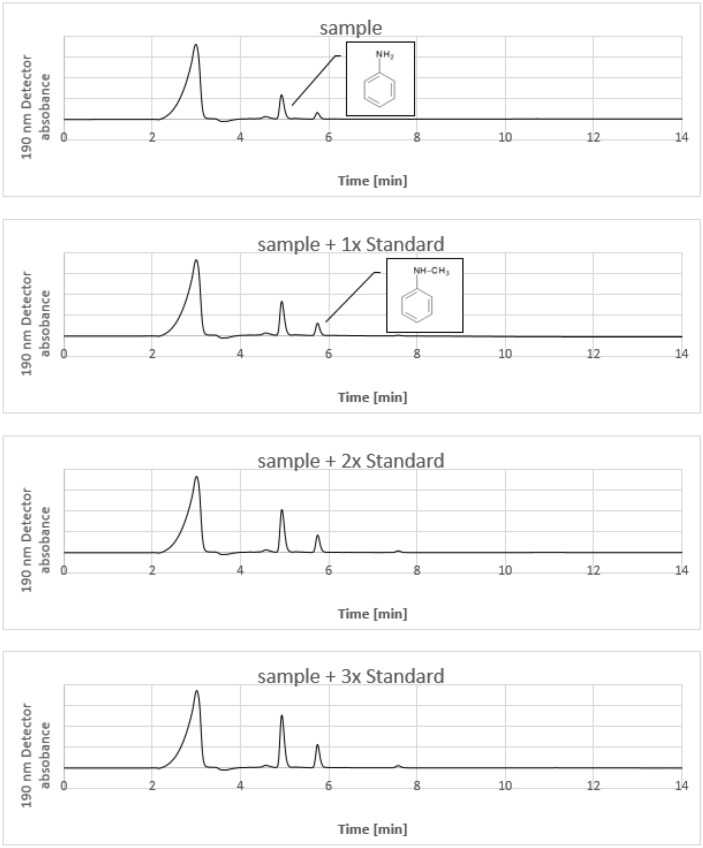
HPLC chromatograms of the acidic indigo extraction without and with the addition of standard. The peak at 4.94 min corresponds to aniline and the peak at 5.76 min corresponds to N-methylaniline.

**Figure 5:**
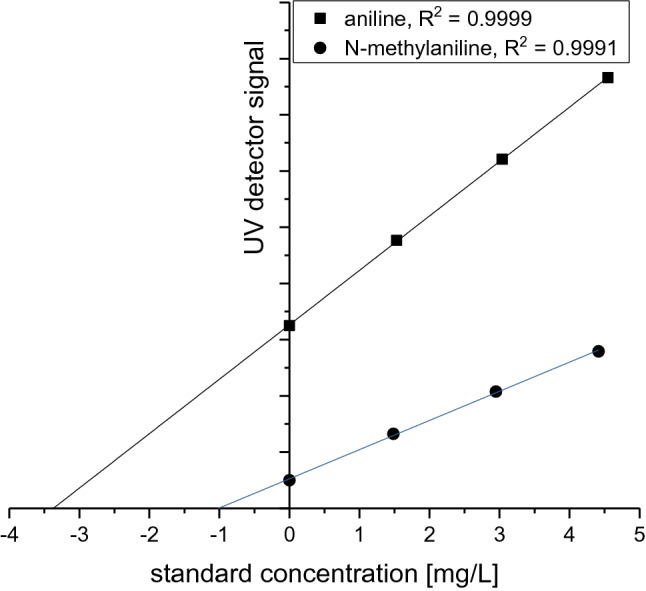
Example for the regression of the linear function of the UV-detector signal versus the standard concentration of the diluted sample solution. The intercept of the regression line with the X-axis determines the sample concentration in the diluted solution.

The washing of indigo with acidic aqueous solution does not dissolve the indigo and the acidic extract of solid indigo contains no aniline and N-methylaniline and leads to the conclusion, that these substances are incorporated in the indigo crystal during indigo formation. Indigo can be converted to leucoindigo by reduction, which is soluble in alkaline water. Therefore it seemed promising to perform the analysis with an extract of the leucoindigo solution, because this would eliminate the possibility of incomplete extraction due to an incorporation in the indigo crystal. It was shown by our investigations, that an incorporation in the indigo crystal is also prevented during the oxidation of a diluted leucoindigo solution to indigo. Obviously aniline and N-methylaniline remain in solution during the oxidation and formation of the indigo crystals. For this purpose 0.3 mL of leucoindigo solution (which corresponds to an indigo concentration of 30% by weight) was transferred into 100 ml 0.01 M HCl solution. This solution was then stirred strongly for 30 min and as a consequence the leucoindigo was oxidized to indigo. The solution was separated from the precipitated indigo with a filter syringe and was used to prepare a series with increasing amount of added standard. Aniline and N-methylaniline show a strong absorption at a wave length of 190 nm and therefore the quantification was performed under this condition. An acetonitrile–water mixture with a volume fraction of 70:30 was used as eluent and the substance separation was performed with a reversed phase silica-C18 column. Figure 4 shows the HPLC chromatograms of the sample and the samples with increasing amount of added standard. The retention time of aniline was 4.94 min and that of the N-methylaniline was 5.76 min and so the two peaks are fully resolved. The large peak at 2.98 min corresponds to the acidic solution and is not the result of a substance, which was extracted from the Indigo solution. The chromatogram also shows, that besides the two substances aniline and N-methylaniline no other substances with considerable amount were extracted from the indigo solution. Figure 5 shows a regression curve by plotting the standard concentration versus the UV detector signal. The coefficients of determination R^2^ in Fig. 5 are better than 0.999 and show, that this quantification method is very accurate. Several samples were analysed with this method and the results are shown in Table 1. The aniline concentration in indigo varies between 0.3 and 0.6% and that of N-methylaniline varies between 0.1 and 0.3% and is therefore very similar to results, which were reported in the literature^24^. A higher aniline concentration is always accompanied by a higher concentration of N-methylaniline, which can be regarded therefore as an indication, that both substances are generated by a similar degradation process during the synthesis of indigo. It is worth to note, that the concept of this analytical method is based on the assumption, that no aniline and N-methylaniline is incorporated into the indigo crystal during its formation in the diluted solution. To confirm this, a second acidic extraction was performed. For this purpose, the precipitated indigo from the first analysis was taken and was then reduced to leucoindigo with sodium dithionite in alkaline water. A measured amount of the leucoindigo solution was then oxidized to indigo in a 0.01 M HCl acidic solution, followed by removing of the precipitated indigo and the clear solution was used to perform the analysis of aniline and N-methylaniline. The results of the second acidic extraction are shown in Table 2. The measured aniline concentration is now in the range of 0.01% and that of N-methylaniline is around 0.007% (in relation to the indigo weight) and so the concentration are considerably reduced by a factor between 30 and 60. This shows clearly, that the acidic extraction of aniline and N-methylaniline from indigo in form of the leucoindigo solution works fine and leads to reliable values.

**Table 1:** Aniline and N-methylaniline concentration of different indigo samples. The percentages by weight are referenced to the indigo weight.

Sample	Aniline (%)	N-methylaniline (%)
1A	0.41	0.11
2A	0.32	0.090
3A	0.35	0.067
4A	0.43	0.19
5A	0.45	0.22
6A	0.53	0.22
7A	0.29	0.083
8A	0.64	0.23

**Table 2:** Aniline and N-methylaniline concentration after the second acidic extraction of different indigo samples. The percentages by weight are referenced to the indigo weight.

Sample	Aniline (%)	N-methylaniline (%)
1B	0.011	0.0072
2B	0.010	0.0075

The acidic extraction with varying concentration of HCl of solid indigo-powder, as well as the extraction with ethanol, combined with ultrasonic treatment, did not remove aniline and N-methylaniline from the indigo crystal (this was shown by the analysis of these solutions with HPLC), which led therefore to the conclusion, that these substances are firmly bound in the crystal. These results are consistent with reports from the literature^24^.

Aniline and N-methylaniline can also be removed from a leucoindigo-solution by extraction with toluene. After that, the amount of these substances can be determined with the analytical approach, presented here. For this purpose, these substances were extracted from the toluene phase into a 0.01 M HCl acidic solution. Due to the decreased pH value of the acidic solution, the transfer of aniline and N-methylaniline into the water phase occurs almost quantitatively.

Table 3 shows the concentration of the two substances in toluene after the acid extraction. The extraction to the acidic solution proceeds almost quantitatively, which was confirmed by a second extraction, showing a considerably decrease of the concentration of aniline and N-methylaniline (see Table 4).

**Table 3:** Aniline and N-methylaniline in toluene after the extraction of the leucoindigo solution. The concentrations are referenced to the toluene solution.

Sample	Aniline (ml/L)	N-methylaniline (ml/L)
1C	0.80	0.33
2C	1.96	1.20

**Table 4:** Aniline and N-methylaniline concentration after the second extraction of the toluene solution. The concentration is referenced to the toluene solution.

Sample	Aniline (ml/L)	N-methylaniline (ml/L)
1D	0.031	0.018

**Figure 6:**
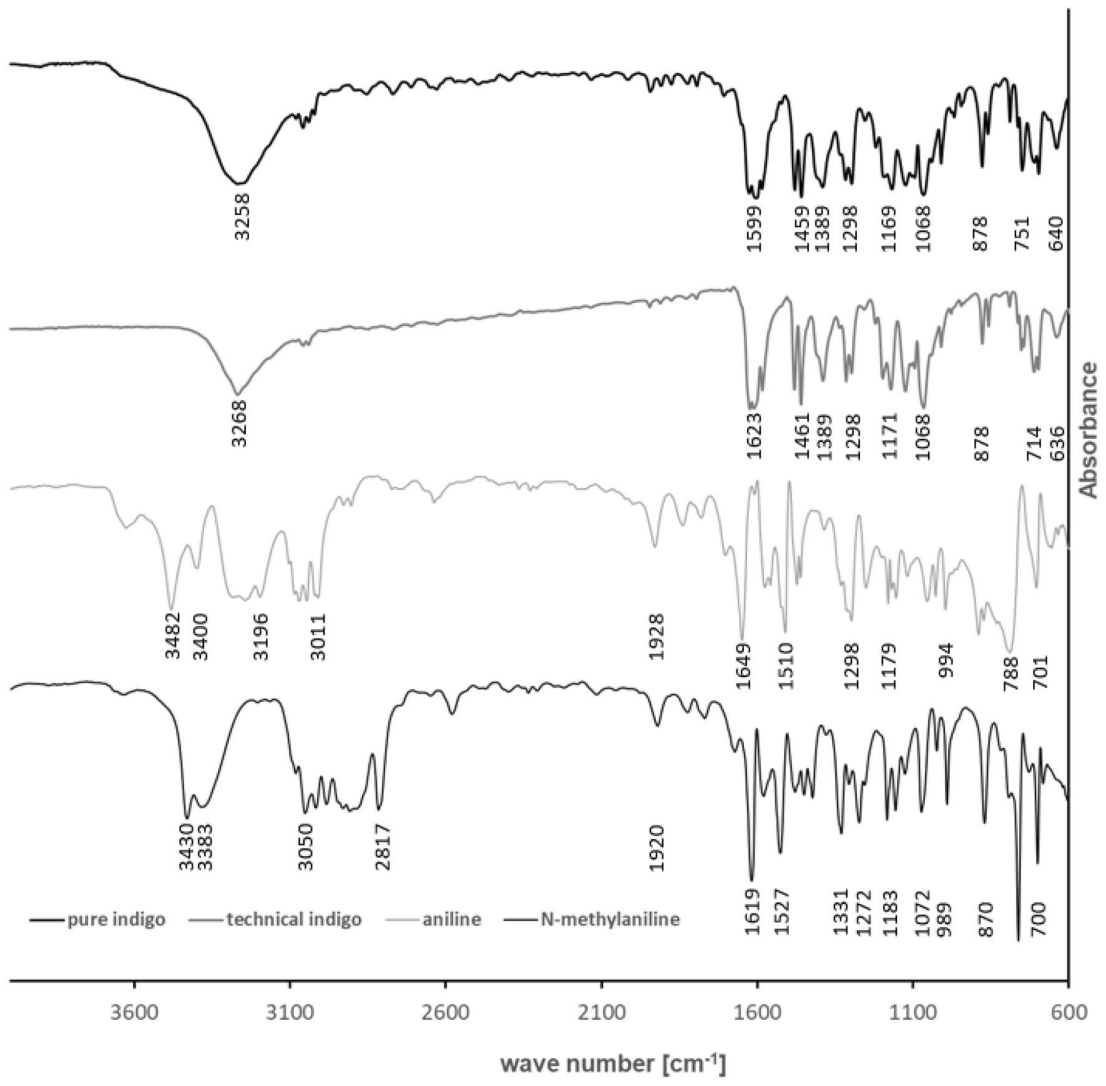
IR spectra of indigo pure, technical indigo (0.63% aniline and 0.30% N-methylaniline), aniline and N-methylaniline. All IR-spectra were measured in the reflection mode. Aniline has a N–H stretching vibration at 3482 cm^−1^ and N-methylaniline at 3431 cm^−1^, indigo does not have this vibration at the corresponding wave number.

The presented results indicate strongly that aniline and N-methylaniline are incorporated into the forming indigo-crystal during its synthesis and therefore a direct detection (without a dissolution of the indigo crystals) is not possible. The main part of incorporated aniline and N-methylaniline can be located either in the crystal or on the surface of the crystals. If aniline and N-methylaniline just adhere weakly to the crystal surface, they should be easily removed by an acidic extraction method. As the majority of both substances, however, cannot be removed by this extraction, we assume that aniline and N-methylaniline are located inside the indigo crystals. Yet, another possible explanation could be that the majority of these substances are very strongly attached at the crystal surfaces. In order to clarify this assumption, indigo powder containing 0.63% aniline and 0.30% N-methylaniline was investigated using FTIR. The FTIR measurement was operated in the reflection mode, which is a surface-sensitive analytical method. If aniline and N-methylaniline are preferably located at the crystal surface, then the FTIR should give a clear indication. The corresponding FTIR spectra of almost pure and technical indigo are shown in Fig. 6. A band assignment of indigo can be found in the literature^18^. The spectra show the typical absorption bands of indigo. A clear distinction between indigo and aniline is possible by the strong N–H stretch vibration of aniline at a wave number of 3482 cm^−1^, whereas no corresponding absorption band in the FTIR spectrum of the indigo sample can be found. A thick shell of aniline and N-methylaniline around the indigo crystal would also suppress the typical absorption bands of indigo, which is not given in this case. The results can be therefore regarded as a clear indication, that aniline is mainly located inside the indigo crystal and therefore a complete quantification needs the dissolution of the crystals.

A possible adsorption of aniline was also investigated by the treatment of indigo with an aniline solution. Indigo powder was stirred for one hour in a solution, which contains 100 ppm aniline. Afterwards the aniline concentration of the solution was analysed with HPLC. According to HPLC investigation, the aniline content was 0.6% (in relation to the indigo) and so there was no significant change in the aniline concentration , indicating that there is no significant amount of aniline adsorbed on the solid indigo crystal surface. This result is a further strong indication, that aniline and N-methylaniline are mainly incorporated in the indigo crystal and only insignificant quantities are adsorbed at the indigo crystal surface. Thus, a removal of these substances seems only to be possible by the dissolution of the crystal structure of indigo.

## Conclusion

Due to the extremely high production volume of indigo dyed products for jeans this technical process requires particular attention with regard to release of contaminants into waste water streams. Commercially synthesized indigo contains up to 0.6% aniline and up to 0.4% N-methylaniline by weight. Due to the negative influence of these substances on the health of people, it is important to develop efficient cleaning processes to remove these contaminants. Therefore commercially suppliers of indigo have a large interest to offer aniline- and N-methylaniline-free indigo to their customers. A powerful and reliable analytical method is the basis for the evaluation of the different cleaning methods. Reports in the literature indicate, that the predominant method to quantify aniline and N-methylaniline in indigo is the photometric analytical technique. However, this technique has significant disadvantages, as the indigo dye extract also contains other substances, which can absorb at the chosen wavelength of measurement. Therefore it is beneficial to separate the different substances with an HPLC column, preventing an overlap of the different substances in the photometric quantification.

Particular attention must be paid to the fact, that aniline and N-methylaniline are incorporated into forming indigo crystals. As these substances seem not to be concentrated at the crystal surface, extraction attempts of solid indigo are difficult. A solution to this problem is the transformation of solid indigo into soluble leucoindigo by reduction. As a consequence aniline and N-methylaniline are separated from the indigo and can be detected using HPLC.

The analytical method presented permits a rapid assessment of the two major contaminants in indigo namely aniline and N-methylaniline, which thus forms a central instrument to control and reduce the release of these toxic substances into the environment. The analytical method presented here can be an important basis for the development and assessment of an industrial feasible method to produce indigo with reduced adverse effect on the human health, especially at a time when green chemistry becomes more and more important.
